# Deep learning for [^18^F]fluorodeoxyglucose-PET-CT classification in patients with lymphoma: a dual-centre retrospective analysis

**DOI:** 10.1016/S2589-7500(23)00203-0

**Published:** 2023-12-21

**Authors:** Ida Häggström, Doris Leithner, Jennifer Alvén, Gabriele Campanella, Murad Abusamra, Honglei Zhang, Shalini Chhabra, Lucian Beer, Alexander Haug, Gilles Salles, Markus Raderer, Philipp B Staber, Anton Becker, Hedvig Hricak, Thomas J Fuchs, Heiko Schöder, Marius E Mayerhoefer

**Affiliations:** Department of Electrical, Engineering, Chalmers, University of Technology, Gothenburg, Sweden; Department of Radiology, Memorial Sloan Kettering, Cancer Center, New York, NY, USA; Department of Radiology, Memorial Sloan Kettering, Cancer Center, New York, NY, USA; Department of Radiology, NYU, Langone Health, Grossman School of Medicine, New York, NY, USA; Department of Electrical, Engineering, Chalmers, University of Technology, Gothenburg, Sweden; Hasso Plattner Institute for Digital Health, Mount Sinai Medical School, New York, NY, USA; Department of AI and Human Health, Icahn School of Medicine at Mount Sinai, New York, NY USA; Department of Radiology, Memorial Sloan Kettering, Cancer Center, New York, NY, USA; Department of Radiology, Memorial Sloan Kettering, Cancer Center, New York, NY, USA; Department of Radiology, Memorial Sloan Kettering, Cancer Center, New York, NY, USA; Department of Biomedical Imaging and Image-guided Therapy; Department of Biomedical Imaging and Image-guided Therapy; Department of Medicine, Memorial Sloan Kettering Cancer Center, New York, NY, USA; Weill Cornell Medical College, Cornell University, New York, NY, USA; Department of Medicine I Medical University of Vienna, Vienna, Austria; Department of Medicine, Memorial Sloan Kettering Cancer Center, New York, NY, USA; Department of Radiology, Memorial Sloan Kettering, Cancer Center, New York, NY, USA; Weill Cornell Medical College, Cornell University, New York, NY, USA; Department of Radiology, NYU, Langone Health, Grossman School of Medicine, New York, NY, USA; Department of Radiology, Memorial Sloan Kettering, Cancer Center, New York, NY, USA; Weill Cornell Medical College, Cornell University, New York, NY, USA; Hasso Plattner Institute for Digital Health, Mount Sinai Medical School, New York, NY, USA; Department of AI and Human Health, Icahn School of Medicine at Mount Sinai, New York, NY USA; Department of Radiology, Memorial Sloan Kettering, Cancer Center, New York, NY, USA; Weill Cornell Medical College, Cornell University, New York, NY, USA; Department of Radiology, Memorial Sloan Kettering, Cancer Center, New York, NY, USA; Department of Biomedical Imaging and Image-guided Therapy; Weill Cornell Medical College, Cornell University, New York, NY, USA; Department of Radiology, NYU, Langone Health, Grossman School of Medicine, New York, NY, USA

## Abstract

**Background:**

The rising global cancer burden has led to an increasing demand for imaging tests such as [^18^F]fluorodeoxyglucose ([^18^F]FDG)-PET-CT. To aid imaging specialists in dealing with high scan volumes, we aimed to train a deep learning artificial intelligence algorithm to classify [^18^F]FDG-PET-CT scans of patients with lymphoma with or without hypermetabolic tumour sites.

**Methods:**

In this retrospective analysis we collected 16 583 [^18^F]FDG-PET-CTs of 5072 patients with lymphoma who had undergone PET-CT before or after treatment at the Memorial Sloa Kettering Cancer Center, New York, NY, USA. Using maximum intensity projection (MIP), three dimensional (3D) PET, and 3D CT data, our ResNet34-based deep learning model (Lymphoma Artificial Reader System [LARS]) for [^18^F]FDG-PET-CT binary classification (Deauville 1–3 *vs* 4–5), was trained on 80% of the dataset, and tested on 20% of this dataset. For external testing, 1000 [^18^F]FDG-PET-CTs were obtained from a second centre (Medical University of Vienna, Vienna, Austria). Seven model variants were evaluated, including MIP-based LARS-avg (optimised for accuracy) and LARS-max (optimised for sensitivity), and 3D PET-CT-based LARS-ptct. Following expert curation, areas under the curve (AUCs), accuracies, sensitivities, and specificities were calculated.

**Findings:**

In the internal test cohort (3325 PET-CTs, 1012 patients), LARS-avg achieved an AUC of 0·949 (95% CI 0·942–0·956), accuracy of 0·890 (0·879–0·901), sensitivity of 0·868 (0·851–0·885), and specificity of 0·913 (0·899–0·925); LARS-max achieved an AUC of 0·949 (0·942–0·956), accuracy of 0·868 (0·858–0·879), sensitivity of 0·909 (0·896–0·924), and specificity of 0·826 (0·808–0·843); and LARS-ptct achieved an AUC of 0·939 (0·930–0·948), accuracy of 0·875 (0·864–0·887), sensitivity of 0·836 (0·817–0·855), and specificity of 0·915 (0·901–0·927). In the external test cohort (1000 PET-CTs, 503 patients), LARS-avg achieved an AUC of 0·953 (0·938–0·966), accuracy of 0·907 (0·888–0·925), sensitivity of 0·874 (0·843–0·904), and specificity of 0·949 (0·921–0·960); LARS-max achieved an AUC of 0·952 (0·937–0·965), accuracy of 0·898 (0·878–0·916), sensitivity of 0·899 (0·871–0·926), and specificity of 0·897 (0·871–0·922); and LARS-ptct achieved an AUC of 0·932 (0·915–0·948), accuracy of 0·870 (0·850–0·891), sensitivity of 0·827 (0·793–0·863), and specificity of 0·913 (0·889–0·937).

**Interpretation:**

Deep learning accurately distinguishes between [^18^F]FDG-PET-CT scans of lymphoma patients with and without hypermetabolic tumour sites. Deep learning might therefore be potentially useful to rule out the presence of metabolically active disease in such patients, or serve as a second reader or decision support tool.

## Introduction

The global cancer burden is rising,^1^ and with it, the demand for imaging tests to accurately assess the extent of disease. For example, the Organization for Economic Co-operation and Development’s data show a 33·5% increase in PET scans between 2015 and 2020, based on 21 countries that provided data for both years.^2^ However, the number of diagnostic imaging specialists, especially those trained in nuclear medicine, remains low, particularly in low-income and middle-income countries,^3^ as evidenced by International Atomic Energy Agency data.^4^ This situation has led to an increasing workload for imaging specialists, which might delay scan reporting and appropriate patient management.^3^

Artificial intelligence (AI) with deep neural networks (DNNs) might be able to better deal with high scan volumes while maintaining or increasing diagnostic accuracy and confidence, and to potentially provide results more rapidly.^5^ AI-based image analysis has shown potential in patient triage, as a decision support tool, and as a second reader,^6–8^ especially when applied to time-consuming, routine tasks. In oncological imaging, one such task is the detection of hypermetabolic tumour sites on 2-deoxy-2-[^18^F]fluorodeoxyglucose ([^18^F]FDG)-PET, a test that is critically important for the diagnostic examination of many cancers. Because increased [^18^F]FDG uptake is observed not only in tumour tissue, but also physiologically in areas such as the urinary tract and myocardium, in various benign conditions,^9^ and secondary to some treatments,^10,11^ PET interpretation is a task that requires specialty expertise.

Here, we describe the development and evaluation of a DNN algorithm that automatically classifies [^18^F]FDG-PET-CT scans of patients with lymphoma. We chose lymphoma as a model disease because [^18^F]FDG-PET-CT is the test of choice for lymphoma according to guidelines, and because the five-point Lugano (Deauville) score provides a well-established reference standard for distinguishing between hypermetabolic and non-hypermetabolic tumour sites, which is essential for patient management.^12,13^ We aimed to train our DNN algorithm Lymphoma Artificial Reader System (LARS) on a large sample of weakly labelled [^18^F]FDG-PET-CT scans of patients treated at a cancer centre in the USA and to measure the accuracy, sensitivity, and specificity for classification of scans with or without hypermetabolic tumour sites in internal and external test cohorts.

## Methods

### Study design and patients

**Figure 1:**
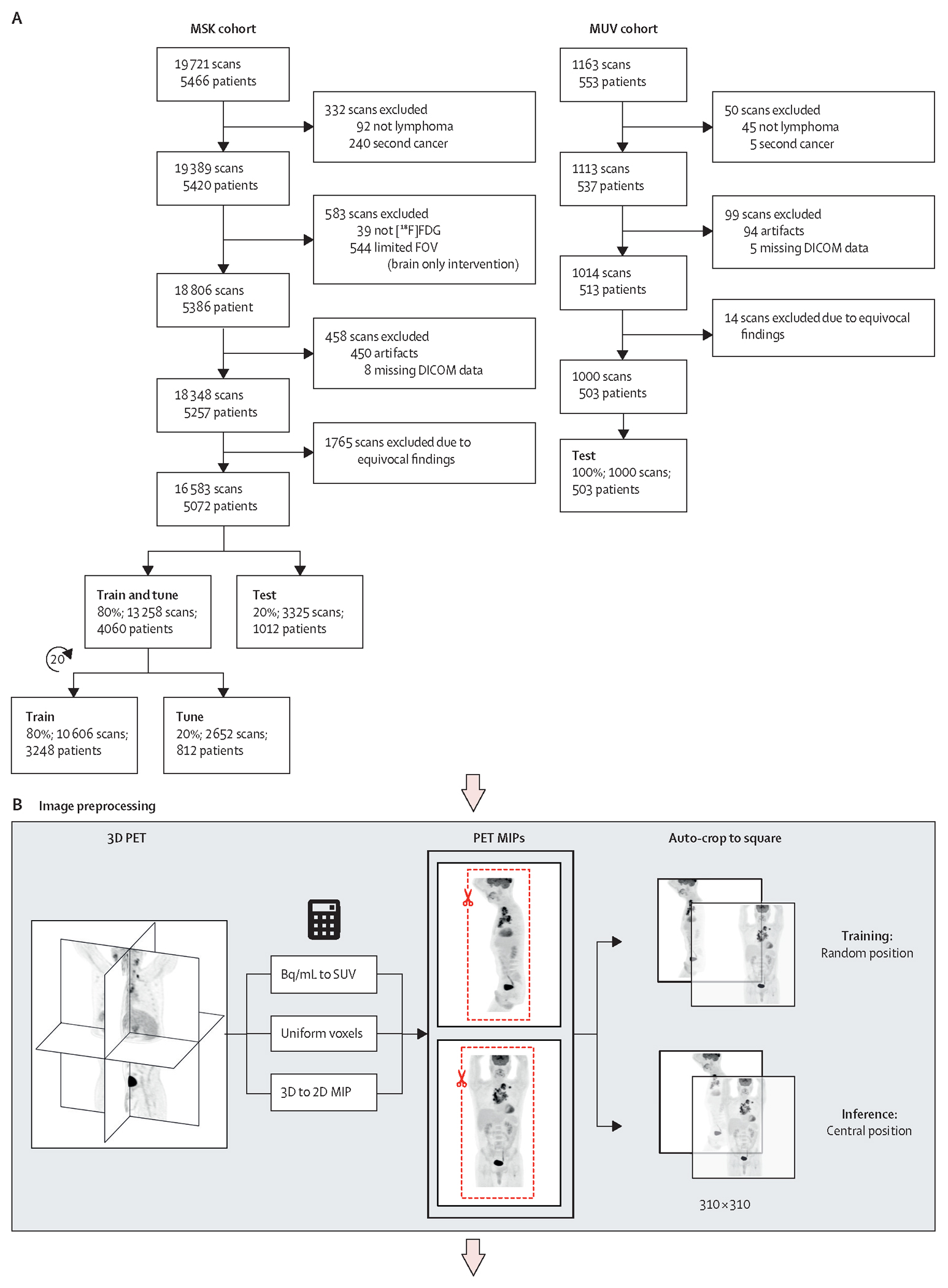

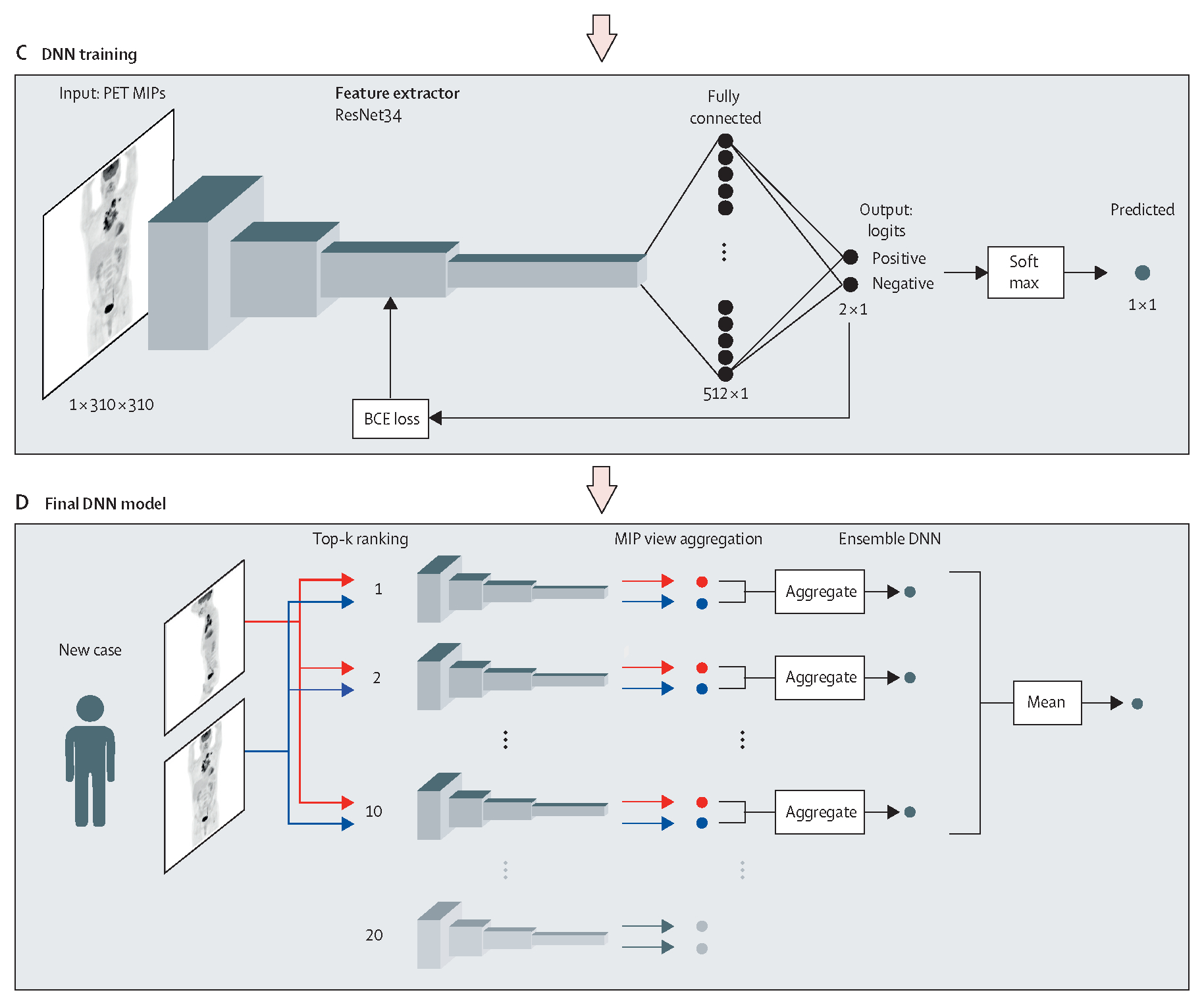
Data and deep learning model (A) The final MSK cohort included 16 583 [^18^F]FDG-PET-CTs. For training, the training and tuning cases were randomly re-split 20 times; each split resulted in a separate trained model. The external MUV test cohort included 1000 [^18^F]FDG-PET-CTs. (B) For the MIP-based model variants (LARS-avg and LARS-max) shown here, each 3D PET image stack was pre-processed to two 2D SUV MIP images (one coronal and one sagittal) each used as a one channel network input. (C) Image features were extracted using a ResNet34 and were then fed to the classifier, which output probabilities for the presence of hypermetabolic tumour sites. Predictions for individual MIP views were aggregated to a single scan-level prediction. (D) The final output during inference was an ensemble of the top-ten performing models from the 20 data splits. BCE=binary cross entropy. DICOM=digital imaging and communications in medicine. FDG=fluorodeoxyglucose. FOV=field of view. LARS=Lymphoma Artificial Reader System. MIP=maximum intensity projection. MSK=Memorial Sloan Kettering Cancer Center. MUV=Medical University of Vienna. SUV=standardised uptake value.

In this retrospective dual-centre study at the Memorial Sloan Kettering Cancer Center (MSK), New York, NY, USA, and the Medical University of Vienna (MUV), Vienna, Austria, patients with biopsy-proven [^18^F]FDG-avid lymphomas according to the Lugano guideline,^12,13^ who had undergone whole-body [^18^F]FDG-PET-CT for routine purposes (staging and treatment response assessment) were eligible for inclusion. The study was approved by the Institutional Review Board of MSK and the Ethics Committee of MUV; informed patient consent was waived. At MSK, the clinical database was queried for patients with the four most common [^18^F]FDG-avid subtypes (Hodgkin lymphoma, diffuse large B-cell, follicular lymphoma, and mantle cell lymphoma) who had undergone PET-CT between Jan 1, 2010, and Jan 31, 2021. At MUV, the centre providing external test data, a list-based search for patients with [^18^F]FDG-avid lymphomas was performed by two clinicians (MR and PBS), aiming for 500 or more patients. At both institutions, exclusion criteria were other cancers in addition to lymphoma; non-[^18^F]FDG-avid or variably [^18^F]FDG-avid lymphomas for which [^18^F]FDG-PET-CT is not recommended;^12,13^ central nervous system lymphoma; blood glucose concentrations greater than 200 mg/dL at PET imaging; non-standard PET-CT (eg, radiotracers other than [^18^F]FDG or single-region PET); and major image artifacts or cropped field-of-view (figure 1). Pre-therapeutic and post-therapeutic PET-CTs of a patient were regarded as independent cases for DNN classification; imaging protocols are in the appendix (pp 2–3). The MSK scans were randomly split on a patient level into 80% training data and 20% held-out test data.

### Procedures

In accordance with the clinical standard of care, [^18^F]FDG-PET scans were rated as positive for hypermetabolic tumour sites when one or more lesions showed uptake higher than the liver (Deauville score 4–5), or otherwise negative (Deauville score 1–3), as determined by a board-certified PET-trained radiologist or nuclear medicine physician specialising in lymphoma (MEM, DL, SC, HZ, or MA). Raters had access to clinical reports and full PET-CT examinations, including maximum intensity projections (MIP). For equivocal findings (eg, tumour *vs* infection or inflammation), previous and follow-up imaging, clinical and laboratory data, and correlative biopsy results were considered, meaning that, while primarily relying on Deauville scores assigned by radiologists, a composite reference standard was used as ground truth. Cases that remained unresolved (eg, due to absence of biopsy or follow-up imaging) were excluded (figure 1). To minimise label noise, the internal MSK test cases (20% of the PET-CTs) and external MUV test cases were curated by the most senior rater (MEM).

We trained LARS on the internal training dataset (80% of MSK scans) to classify PET-CTs as positive (=1) or negative (=0) for hypermetabolic tumour sites. The model consisted of an image feature extractor and a classifier (figure 1; appendix p 6). We used a ResNet34 convolutional neural network for feature extraction;^14^ further details are n the appendix (pp 4–5). To reduce generalisation error and increase model performance, an ensemble model was used for the final scan classification.

Seven DNN variants were constructed: (1) LARS-avg, a classification based on the average (mean) probability of coronal and sagittal two dimensional (2D) PET MIP images, aiming for high accuracy; (2) LARS-max, a classification based on the maximum probability of coronal and sagittal 2D PET MIP images, aiming for high sensitivity; (3) LARS-pt3d, a classification based on the probability of the three dimensional (3D) PET volume; (4) LARS-ct, a classification based on the probability of the 3D CT volume; (5) LARS-ptct, a classification based on the probability of the 3D PET-CT volume; (6)LARS-avg-ct, a classification based on the probability of the separately trained LARS-avg aggregated with the probability of LARS-ct; and (7) LARS-max-ct, a classification based on the probability of the separately trained LARS-max aggregated with the probability of LARS-ct. We used a 2D ResNet (21 million parameters) to analyse MIPs, and a 3D ResNet (63 million parameters) for the 3D image volumes.

For LARS-avg and LARS-max, probabilities calculated for individual MIP views were aggregated to provide a single probability per scan; the trained model was the same, but prediction aggregation differed. Models were trained using a binary cross-entropy loss function; hyperparameters were tuned using the best tuning dataset area under the curve (AUC). Separately for each LARS variant, the top-ten ensemble mean was calculated based on aggregated probabilities (appendix p 4). It took around 32 ms to classify a single MIP image using the 2D LARS models.

### PET classification and statistical analysis

LARS variants were tested on the previously held-out internal MSK test dataset (20% of MSK PET scans), and, separately on the external MUV dataset. Receiver operating characteristic (ROC) curves were constructed, and the AUC, sensitivity, specificity, and balanced accuracy for classification of PET-CT scans with or without hypermetabolic tumour sites were calculated at the optimal threshold determined by Youden’s index.^15^ 95% CIs were calculated as the 2·5th and 97·5th percentile of 1000 bootstraps of the scans. For LARS-avg, misclassified cases were assigned to one of six groups by a rater (MEM): (1) human labelling error upon review; (2) infection or inflammation (eg, pneumonia or osteoarthritis); (3) iatrogenic (eg, bone marrow repopulation following granulocyte colony-stimulating factor treatment, bowel uptake following metformin, post-treatment thymic rebound, tracer extravasation, catheter-related uptake, or biopsy or surgery-related uptake); (4) metabolically active brown fat; (5) other (eg, tracer contamination or muscle activity); or (6) unknown. To better interpret misclassified cases and highlight image regions responsible for prediction, we applied Gradient-weighted Class Activation Mapping (Grad-CAM)^16^ and studied mean heatmaps of the top-ten ensemble models. Finally, to investigate the extracted image features in more detail, we reduced the dimensionality of the 512-dimensional features space to two, using uniform manifold approximation and projection (UMAP),^17^ and visualised the reduced feature space.

To evaluate the impact of the training dataset size, and the impact of integration of clinical data into the models, on classification results, we performed dedicated experiments (appendix pp 12–13).

### Role of the funding source

The funder of the study had no role in study design, data collection, data analysis, data interpretation, or writing of the report.

## Results

19721 PET-CT scans of 5466 patients with the four most common [^18^F]FDG-avid subtypes (Hodgkin lymphoma, diffuse large B-cell, follicular lymphoma, and mantle cell lymphoma) who had undergone PET-CT between Jan 1, 2010, and Jan 31, 2021, were in the MSK database. After exclusions, the MSK dataset comprised 16 583 [^18^F]FDG-PET-CT scans of 5072 patients; 13 258 (80%) scans were used as training and tuning dataset, and 3325 (20%) scans were used as an internal test dataset (figure 1). After exclusions, the external MUV test dataset comprised 1000 [^18^F]FDG-PET-CT scans of 503 patients. Demographic, pathological, and PET-CT details are in table 1 and the appendix (p 3), and the distribution of Deauville scores and respective model accuracies are in the appendix (p 7).

**Figure 2:**
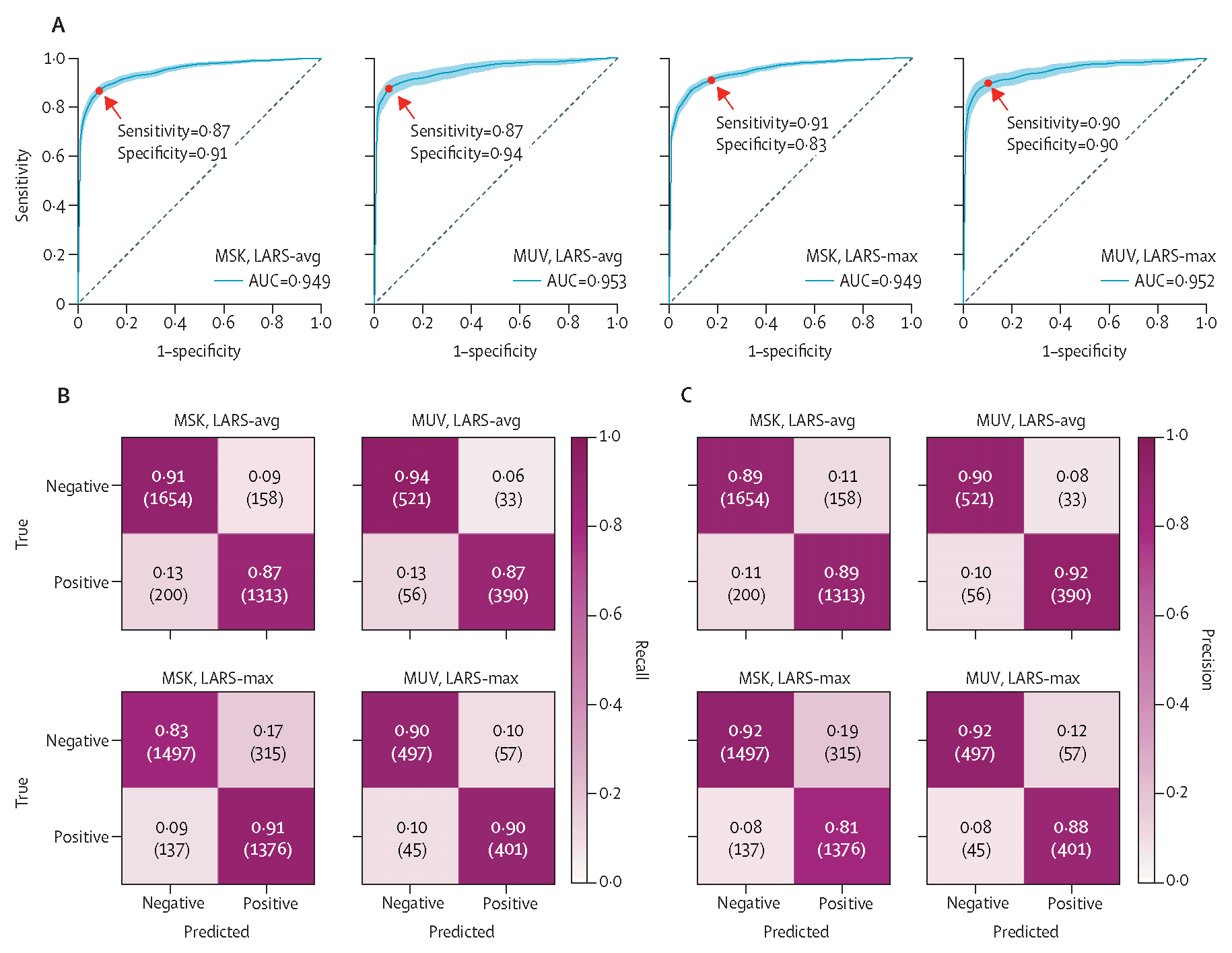
Receiver operating characteristic curves for the classification of PET-CTs with or without hypermetabolic tumour sites in the held-out internal MSK test dataset, and the external MUV test dataset, using the MIP-based model variants LARS-avg and LARS-max (A) AUC for both cohorts were 0·95. Sensitivities and specificities at the Youden index (determined by MSK LARS-avg) are shown; shaded areas represent 95% CIs of 1000 bootstraps. Recall (B) and precision confusion matrices (C) for LARS-avg and LARS-max are shown for the internal MSK and the external MUV test dataset (absolute numbers in parentheses). LARS=Lymphoma Artificial Reader System. LARS-avg=classification based on mean probability of coronal and sagittal 2D PET MIP images. LARS-max=classification based on maximum probability of coronal and sagittal 2D PET MIP images. MIP=maximum intensity projection. MSK=Memorial Sloan Kettering Cancer Center. MUV=Medical University of Vienna.

**Figure 3:**
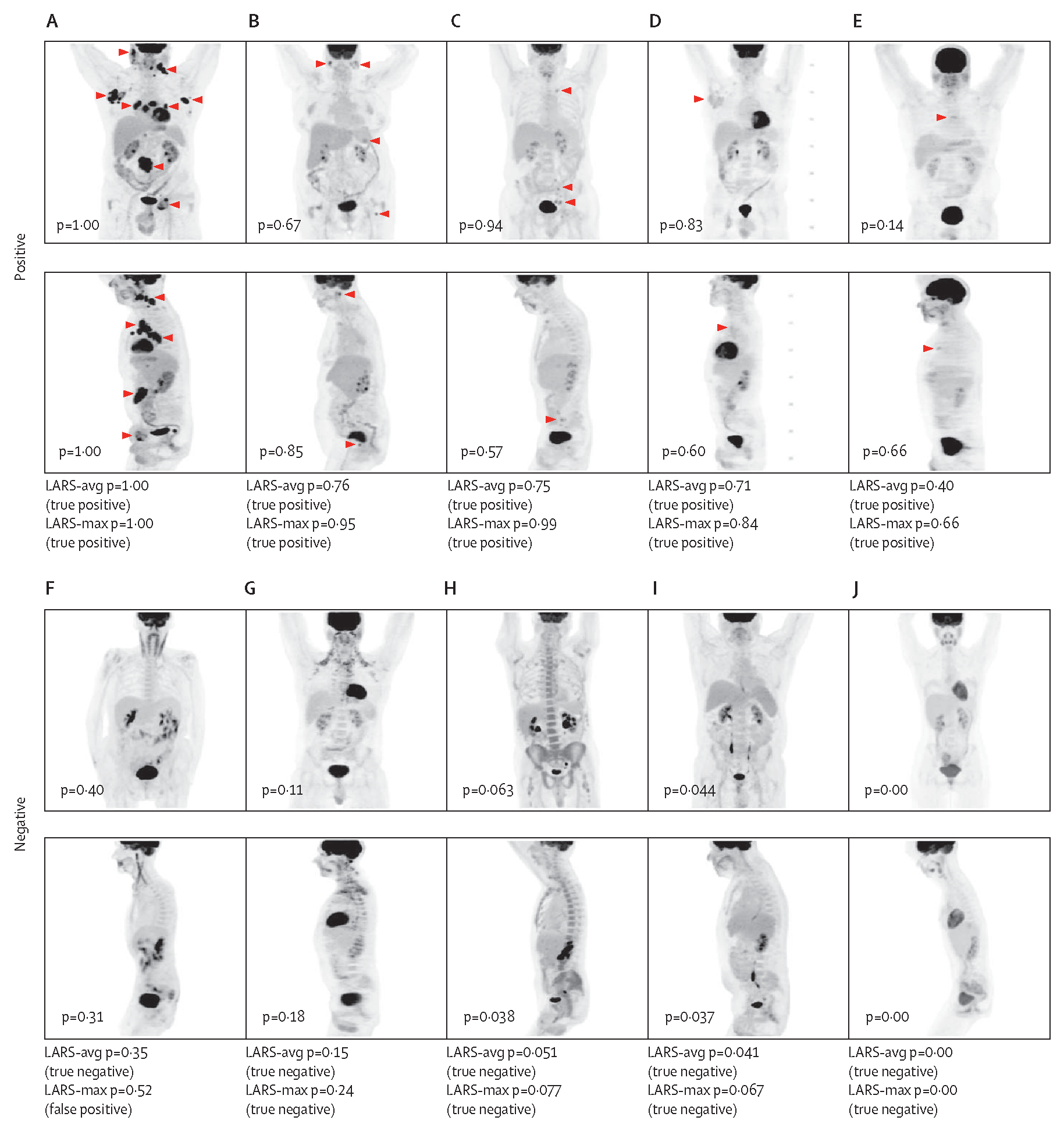
Examples of true-positive and true-negative PET scans (SUV range 1–10) in the MSK test cohort, including ensemble probabilities and classification results for the MIP-based model variants LARS-avg and LARS-max Each MIP view is labelled with the mean probability of that view over the top-ten models. The ensemble probability threshold was set to 0·3614, determined by the Youden index of MSK LARS-avg. Hypermetabolic tumour sites on true-positive scans are marked with arrowheads (A–E). PET-negative cases were correctly predicted despite different patterns of physiologic uptake, such as cervical muscles (F), brown fat (G), bone marrow activation (H), marked focal FDG excretion via ureters as well as esophagitis (I), and focal bowel uptake (F, J). FDG=fluorodeoxyglucose. LARS=Lymphoma Artificial Reader System. LARS-avg=classification based on mean probability of coronal and sagittal 2D PET MIP images. LARS-max=classification based on maximum probability of coronal and sagittal 2D PET MIP images. MIP=maximum intensity projection. MSK=Memorial Sloan Kettering Cancer Center.

LARS-avg and LARS-max were the overall best-performing models with AUCs of 0·95 in internal and external test datasets, at the maximum Youden index (table 2; figure 2). LARS-avg achieved an AUC of 0·949 (95% CI 0·942–0·956), accuracy of 0·890 (0·879–0·901), sensitivity of 0·868 (0·851–0·885), and specificity of 0·913 (0·899–0·925) in the internal test cohort; and an AUC of 0·953 (0·938–0·966), accuracy of 0·907 (0·888–0·925), sensitivity of 0·874 (0·843–0·904), and specificity of 0·949 (0·921–0·960) in the external test cohort. Recall and precision confusion matrices are shown in figure 2, and examples of correctly classified cases and respective LARS probabilities are shown in figure 3. Of the 358 cases misclassified by LARS-avg in the MSK test dataset, 158 were false-positive; 135 could be explained by uptake unrelated to lymphoma, with infection or inflammation and iatrogenic effects as the dominant causes (appendix p 7). When correcting the labels of the nine cases mislabelled by human error in the MSK test dataset, the AUC of LARS-avg minimally increased (from 0·949 to 0·951). In the MUV dataset, the AUC of LARS-avg also slightly increased (from 0·953 to 0·961) after correction of the seven mislabelled cases. When ROC curve cutoff values were modified to increase sensitivity and specificity to 0·95, the corresponding specificity and sensitivity reduced (table 2). UMAP isolines show that features of true-positive and true-negative cases were well separated (appendix p 9). Grad-CAM heatmaps indicate the pixel-level importance for the prediction (appendix p 10).

When correcting the labels of the nine cases mislabelled by human error in the MSK test dataset, the AUC of LARS-avg minimally increased (from 0·949 to 0·951). In the MUV dataset, the AUC of LARS-avg also slightly increased (from 0·953 to 0·961) after correction of the seven mislabelled cases (table 2; figure 2). Correcting the nine cases mislabelled by human error minimally increased the AUC to 0·951. Similarly, in the external MUV cohort, the AUC of LARS-max slightly improved from 0·952 to 0·960 after correction of the seven human-mislabelled cases. Again, further improvement of sensitivity or specificity to 0·95 through modification of ROC cut-off values resulted in a decrease of the respective specificity and sensitivity (table 2).

LARS-pt3d yielded an AUC of 0·933 in the internal MSK test dataset and 0·921 in the MUV test dataset, based on the LARS-avg Youden index (table 2). By comparison, LARS-ct performance was unsatisfactory with AUCs of 0·682 in MSK, and 0·655 in MUV test datasets. LARS-ptct, the jointly trained 2-channel 3D PET plus CT model, achieved higher AUCs of 0·939 in the MSK test dataset and 0·932 in the MUV test dataset, showing a slight superiority over LARS-pt3d particularly with regard to specificity (0·92 in the MSK and 0·91 in the MUV dataset). The best-performing combination model was LARS-avg-ct, with AUCs of 0·944 for the MSK test dataset and 0·947 for the MUV test dataset, and an overall similar, but not superior, performance relative to LARS-avg (table 2). Although LARS-max-ct achieved the highest sensitivity of all models (0·99–1 in both test datasets), specificity was poor (2–16%; table 2).

DNN model performance improved with an increased training dataset size, from an AUC of 0·74 at 1% of the training data, to 0·95 at 100%, whereas integration of clinical information into the model did not improve results (appendix pp 12–13).

## Discussion

With a sample size of 17 583 [^18^F]FDG-PET-CT scans in 5575 patients with lymphoma, this study is, to our knowledge, currently the largest study to apply deep learning to PET-CT and, by extension, also PET-CT in oncology. The largest previous study to apply deep learning to [^18^F]FDG-PET-CT focused on tumour segmentation and included 3664 scans in patients with lung cancer and lymphoma.^18^ LARS, our DNN algorithm for classification of PET-CT scans with or without hypermetabolic tumour sites, achieved an AUC of 0·95 in both internal and external test datasets, with a balanced accuracy of 87–91% when using PET MIPs as input. These results were achieved despite real-world technical differences between the two centres: while at MSK, virtually all PET-CTs were performed on GE Healthcare device models, and with a fixed radiotracer dosage, PET-CTs at MUV were performed on Siemens devices, and with the radiotracer dosage adapted to patient body mass. Additionally, the MSK cohort included patients with the four most common [^18^F]FDG-avid lymphoma subtypes (Hodgkin lymphoma, diffuse large B-cell lymphoma, follicular lymphoma, and mantle cell lymphoma), whereas the MUV cohort also included other [^18^F]FDG-avid subtypes, such as peripheral T-cell lymphoma and Burkitt lymphoma. LARS might, therefore, be able to generalise and yield robust performance across centres, scanners, and histologies, and (although tested exclusively on lymphoma in our study for reference standard consistency) might possibly also be applicable to other FDG-avid cancers.

There are several potential applications of LARS to aid radiologists and nuclear medicine physicians in clinical practice, especially when PET-CT scan volumes are high. First, LARS might be useful to rule out the presence of hypermetabolic tumour sites, for instance in a post-treatment setting. Based on this information, preliminary reports could be generated automatically, for example when high scan volumes prevent imaging specialists from providing the final reports in time (eg, at the time of patients’ visits with clinicians). This approach could possibly reduce delays in management decisions for many patients, allowing imaging specialists to prioritise scans with a higher likelihood of FDG-avid disease. For such an application, an algorithm would require high sensitivity to minimise false-negative predictions. LARS-max, the algorithm version using maximum probability aggregation to achieve a sensitivity of 91%, might be useful in such a scenario. To serve as a true replacement for human readers, however, an even higher sensitivity would probably be desirable in clinical practice, to match the performance levels of human experts. For the latter however, few comparative data exist at present. Alotaibi and colleagues analysed 4099 PET-CT reports generated over an 18-month period at a tertiary care centre, and found that 2·2% contained an addendum that revealed a retrospectively discovered diagnostic error.^19^ The true error rate for PET-CT reports by physicians, which would also include errors that go unnoticed, remains unknown, but is probably higher, given that for radiology tests in general, a human error rate of 3–5% has been estimated.^20^ To achieve comparable sensitivity levels (eg, 95%) with our model, modification of the cutoff value between positive and negative scans, at the expense of specificity, might be an option (table 2).

Another application would be to use LARS as a second reader, to further improve physicians’ accuracy and diagnostic confidence, or help maintain a high accuracy or confidence despite an increased workload. Because such an application requires a balance between good sensitivity and specificity, LARS-avg, with its 87% sensitivity and up to 95% specificity, or alternatively, LARS-ptct or LARS-avg-ct, both of which offered a similar performance, appear to be reasonable choices, and might assist in shortening reading times. Visualisation techniques such as Grad-CAM (appendix p 10) might facilitate integration of such an application into clinical practice.

Finally, due to their high specificities, LARS-avg, or alternatively, LARS-ptct, might also be useful as decision support tools, for instance, in equivocal cases (eg, tumour *vs* infection or inflammation), because these models were not trained using information from merely the current, but also from previous and follow-up scans, and, if available, biopsies.

Previous studies used deep learning for lesion detection or classification,^18,21–23^ segmentation,^18,21–22,24–29^ and outcome prediction or prognostication on [^18^F]FDG-PET.^27,30^ A comprehensive overview of such applications in lymphoma was provided by Hasani and colleagues in 2022.^31^ Contrary to these applications, we focused on a specific, routine task that radiologists and nuclear medicine physicians perform in their day-to-day practice, in a multitude of cancers: distinguishing between PET-CTs with, and those without, hypermetabolic tumour sites, before and after treatment. We believe that this simple task (ie, disease detection on a per-scan, rather than on a per-lesion basis) is the first step towards fully automated AI-based image interpretation, upon which specific applications, such as automated tumour segmentation, response assessment, and outcome prediction or prognostication, can build. To our knowledge, only a single study in a mixed oncological population evaluated a deep learning approach similar to ours for [^18^F]FDG-PET classification. However, in that study, the algorithm was trained to classify PET scans not only as positive or negative, but alternatively, also as equivocal (22% of cases) for hypermetabolic tumour sites,^32^ which might hamper its clinical applicability. Furthermore, no fixed criteria (such as Deauville scores) for PET curation were specified, and no external validation was performed.

We analysed 2D and 3D PET, as well as 3D CT data, to generate and compare different one-modality and two-modality LARS variants. The PET MIP-based models (LARS-avg and LARS-max) allowed us to use a smaller network with fewer parameters, which decreased computation time (classification of a single MIP took only approximately 32 ms), and thereby also energy consumption and carbon footprint. MIPs are commonly included in PET examinations, providing less noisy image representations than 3D volumes and offering an overview of disease status. DNN predictions of the sagittal and coronal MIP views were aggregated to form a case-level prediction. The risk with this approach is that tumour-related uptake may be obscured by other high-uptake structures such as the heart, the urinary tract, and the bowel, occasionally even on both sagittal and coronal views (appendix p 11). We took this into consideration by creating a second DNN version with modified MIP aggregation, which used the maximum probability for FDG-avid tumour sites of the two MIP views (LARS-max), rather than the average (LARS-avg), for classification. LARS-max was therefore designed to increase model sensitivity at the cost of lower specificity, and yielded a sensitivity of 90–91% (*vs* 87% with LARS-avg), while retaining an acceptable specificity of 83–90% (*vs* 91–95% with LARS-avg).

Surprisingly, neither the 3D PET model (LARS-pt3d) nor the different PET-CT models (including the 2-channel 3D PET plus CT model, LARS-ptct) were superior to the PET MIP-based models. Since the same type of DNN architecture (ResNet34) was used for all models (the only difference being the use of 3D convolutions for 3D image data) we hypothesise that the lower image noise on MIPs and the lower model complexity (21 M parameters for both LARS-avg and LARS-max *vs* 63 million for LARS-pt3d) were responsible for our results. The additional 3D CT information within LARS-ptct slightly improved results relative to LARS-pt3d, especially with regard to specificity (table 2). This finding is understandable because several causes for false-positive results on PET, such as brown fat or metformin-related bowel uptake, do not have a CT correlate. Otherwise, the contribution of CT to the performance of our models was limited, probably because for post-treatment scans of FDG-avid lymphomas, tumour site evaluation is based exclusively on [^18^F]FDG-PET findings.^12,13^ Notably, a previous study in lung cancer and lymphoma also suggested that CT adds little to [^18^F]FDG-PET regarding lesion detection and classification.^21^ However, inclusion of CT in DNN models might potentially be of greater importance in other cancers, where PET results are less relevant, or less well-established, than in lymphoma.

Our study has several limitations. Compared with PET-CT scans of healthy individuals (eg, with benign lesions), the PET-negative cases in our study were arguably more difficult to classify correctly due to frequent increased FDG uptake resulting from systemic treatment or interventions (eg, biopsies or lymphadenectomy). The latter reasons for increased FDG uptake might lead to not only false-positives, but also to false-negatives when lesions are obscured by FDG uptake related to treatment or intervention. While these effects probably decreased our model performance, they might also affect human visual interpretation, and reflect clinical reality, because [^18^F]FDG-PET is frequently used after treatment. Therefore, to perform well under real-world conditions, a DNN must learn to distinguish such iatrogenic patterns of [^18^F]FDG uptake from malignancy. The exclusion of a substantial number of unresolved cases from the MSK cohort, for which no biopsy or follow-up imaging were available, means that we might have overstated our models’ performances in this cohort to some degree, because difficult to classify cases might potentially have been omitted. However, because the number of such unresolved, excluded cases was quite low in the MUV cohort, but model performance was very similar to that in the MSK cohort, overestimation of our models’ performances was probably limited. Contrary to many previous studies that focused on a single lymphoma subtype,^22,23,26–30^ our cohort was a mix of Hodgkin and FDG-avid, aggressive, and indolent, B-cell and T-cell non-Hodgkin lymphomas. This mixture clearly made the classification task more difficult, given that patterns of involvement and degree of [^18^F]FDG uptake differ between lymphoma subtypes.^33^ However, this mixture of lymphoma subtypes again reflects the day-to-day work of radiologists and nuclear medicine physicians, and if our algorithm was to be applied to other cancers, complexity would increase even further. Our models were designed for binary classification, rather than for PET-based four category Lugano response classification, so that, in post-treatment scans with hypermetabolic tumour sites, a more granular response assessment by physicians is required. Finally, as classifiers, our models also do not provide pixel-level tumour localisations or direct quantification of tumour volumes. Lacking lesion annotations, LARS could therefore possibly be combined with previously published lymphoma segmentation models.^18^

In conclusion, we have presented a DNN model capable of distinguishing between [^18^F]FDG-PET scans with and without hypermetabolic tumour sites in patients with lymphoma, as a model disease for FDG-avid cancers. Our algorithm could potentially be used to rule out hypermetabolic tumour sites, for instance in a post-treatment setting, and thereby serve as a basis for automatically generated (preliminary) reports. Furthermore, our algorithm might possibly be useful as a second reader or decision-support tool, potentially helping radiologists to deal with high scan volumes. Because our PET MIP-based models were not inferior to 3D PET and CT-based models, they might be a reasonable choice, especially also regarding speed, hardware requirements, and energy consumption. Prospective evaluation of our models is, however, clearly required, to determine whether they can be used to truly rule out hypermetabolic tumour sites on PET-CT in lymphoma and other FDG-avid cancers in clinical practice, and to investigate their impact on reading times, accuracy, diagnostic confidence, and incurred risk.

## Supplementary Material

1

## Figures and Tables

**Table 1:** Patient population characteristics

	MSK cohort (n=16 583 PET-CT scans; n=5072 patients)						MUV cohort test dataset (n=1000 PET-CT scans; n=503 patients)		
	Training and tuning dataset (n=13 258 scans; n=4060 patients)			Test dataset (n=3325 scans; n=1012 patients)			Total	Negative	Positive
	Total	Negative	Positive	Total	Negative	Positive			

PET-CT scans	13 258/16 583 (80%)	6900/13 258 (52%)	6358/13 258 (48%)	3325/16 583 (20%)	1812/3325 (54%)	1513/3325 (46%)	1000/1000 (100%)	554/1000 (55%)	446/1000 (45%)
Median age, (IQR)	58 (38–70)	56 (35–69)	61(42–71)	56 (36–69)	55 (34–68)	59 (38–69)	50 (32–65)	48 (31–63)	50 (33–65)
Sex*									
Male	7705/13 258 (58%)	3895/7705 (51%)	3810/7705 (49%)	1949/3325 (59%)	1053/1949 (54%)	896/1949 (46%)	524/1000 (52%)	282/524 (54%)	242/524 (46%)
Female	5553/13 258 (42%)	3005/5553 (54%)	2548/5553 (46%)	1376/3325 (41%)	759/1376 (55%)	617/1376 (45%)	476/1000 (48%)	272/476 (57%)	204/476 (43%)
Histology†									
Diffuse large B-cell lymphoma	6282/13 258 (47%)	3221/6282 (51%)	3061/6282 (49%)	1560/3325 (47%)	866/1560 (56%)	694/1560 (44%)	263/1000 (26%)	142/263 (54%)	121/263 (46%)
Hodgkin lymphoma	3945/13 258 (30%)	2228/3945 (56%)	1717/3945 (44%)	1043/3325 (31%)	586/1043 (56%)	457/1043 (44%)	381/1000 (38%)	208/381 (55%)	173/381 (45%)
Follicular lymphoma	1628/13 258 (12%)	702/1628 (43%)	926/1628 (57%)	347/3325 (11%)	182/347 (49%)	192/347 (51%)	190/1000 (19%)	94/190 (49%)	96/190 (51%)
Mantle cell lymphoma	1403/13 258 (11%)	749/1403 (53%)	654/1403 (47%)	348/3325 (10%)	178/348 (51%)	170/348 (49%)	32/1000 (3%)	18/32 (56%)	14/32( 44%)
Peripheral T-cell lymphomas, all types	¨	¨	¨	¨	¨	¨	56/1000 (6%)	34/56(61%)	22/56 (39%)
Post transplant lymphoproliferative disease	¨	¨	¨	¨	¨	¨	38/1000 (4%)	31/38 (82%)	7/38(18%)
Burkitt	¨	¨	¨	¨	¨	¨	35/1000 (4%)	23/35 (66%)	12/35 (34%)
Other	¨	¨	¨	¨	¨	¨	5/1000 (1%)	4/5 (80%)	1/5 (20%)

Data are n/N (%) or median (IQR).

* Numbers for sex are shown per scan, rather than per patient.

† Due to transformation in the course of disease of some patients, histologies are shown per scan, rather than per patient.

**Table 2:** LARS performance metrics for MSK and MUV test cohorts

	LARS-avg	LARS-max	LARS-pt3d	LARS-ct	LARS-ptct	LARS-avg-ct	LARS-max-ct

**MSK cohort (3325 PET-CTs; 1012 patients)**							
AUC	0.949 (0.942–0.956)	0.949 (0.942–0.956)	0.933 (0.924–0.941)	0.682 (0.664–0.701)	0.939 (0.930–0.948)	0.944 (0.936–0.952)	0.939 (0.930–0.948)
Sensitivity	0.868 (0.851–0.885)	0.909 (0.896–0.924)	0.827 (0.808–0.846)	0.544 (0.518–0.570)	0.836 (0.817–0.855)	0.872 (0.855–0.890)	0.993 (0.989–0.997)
Specificity	0.913 (0.899–0.925)	0.826 (0.808–0.843)	0.896 (0.882–0.910)	0.703 (0.682–0.723)	0.915 (0.901–0.927)	0.903 (0.889–0.917)	0.161 (0.145–0.178)
Balanced accuracy	0.890 (0.879–0.901)	0.868 (0.858–0.879)	0.865 (0.853–0.877)	0.624 (0.607–0.640)	0.875 (0.864–0.887)	0.888 (0.877–0.898)	0.577 (0.569–0.586)
Sensitivity at 95% specificity	0.820 (0.791–0.848)	0.810 (0.785–0.834)	0.760 (0.730–0.792)	0.250 (0.221–0.278)	0.781 (0.754–0.816)	0.819 (0.792–0.846)	0.794 (0.759–0.824)
Specificity at 95% sensitivity	0.650 (0.600–0.702)	0.650 (0.594–0.705)	0.590 (0.527–0.650)	0.130 (0.113–0.164)	0.610 (0.559–0.669)	0.640 (0.554–0.714)	0.640 (0.548–0.688)
Number of false positives	158	315	188	536	154	175	1521
Number of false negatives	200	137	261	687	247	193	10
**MUV cohort (1000 PET-CTs; 503 patients)**							
AUC	0.953 (0.938–0.966)	0.952 (0.937–0.965)	0.921 (0.904–0.939)	0.655 (0.622–0.687)	0.932 (0.915–0.948)	0.947 (0.936–0.958)	0.936 (0.920–0.951)
Sensitivity	0.874 (0.843–0.904)	0.899 (0.871–0.926)	0.834 (0.801–0.869)	0.841 (0.808–0.874)	0.827 (0.793–0.863)	0.881 (0.860–0.902)	0.998 (0.993–1.000)
Specificity	0.949 (0.921–0.960)	0.897 (0.871–0.922)	0.866 (0.839–0.892)	0.301 (0.264–0.339)	0.913 (0.889–0.937)	0.913 (0.898–0.929)	0.023 (0.012–0.037)
Balanced accuracy	0.907 (0.888–0.925)	0.898 (0.878–0.916)	0.850 (0.829–0.873)	0.571 (0.546–0.595)	0.870 (0.850–0.891)	0.897 (0.884–0.910)	0.511 (0.504–0.518)
Sensitivity at 95% specificity	0.862 (0.819–0.899)	0.858 (0.811–0.898)	0.777 (0.731–0.824)	0.176 (0.128–0.228)	0.786 (0.741–0.830)	0.854 (0.828–0.877)	0.787 (0.715–0.847)
Specificity at 95% sensitivity	0.650 (0.537–0.777)	0.640 (0.535–0.784)	0.470 (0.387–0.596)	0.130 (0.097–0.178)	0.550 (0.367–0.706)	0.600 (0.475–0.710)	0.600 (0.448–0.711)
Number of false positives	33	57	74	387	48	48	541
Number of false negatives	56	45	74	71	77	53	1

Data are performance metrics (95% CI) or n. 95% CIs are from 1000 bootstraps. AUC=area under the curve. LARS=Lymphoma Artificial Reader System.

LARS-avg=classification based on mean probability of coronal and sagittal 2D PET maximum intensity projection images. LARS-max=classification based on maximum probability of coronal and sagittal 2D PET maximum intensity projection images. LARS-pt3d=classification based on probability of the 3D PET volume. LARS-ct= classification based on the probability of the 3D CT volume. LARS-ptct=classification based on the probability of the 3D PET-CT volume. LARS-avg-ct=classification based on the probability of the separately trained LARS-avg aggregated with the probability of LARS-ct. LARS-max-ct=classification based on the probability of the separately trained LARS-max aggregated with the probability of LARS-ct. MSK=Memorial Sloan Kettering Cancer Center. MUV=Medical University of Vienna.

## Data Availability

Upon publication, the data that support the findings of this Article are available for academic purposes upon written request to the corresponding author. The DNN source code and trained models can be downloaded for academic or non-commercial purposes from https://github.com/haeggsti/lymphoma_classification_2023/. Images are not publicly available because they contain sensitive information that could compromise patient privacy; access to these data, therefore, requires a signed data transfer or access agreement, and requests will be reviewed on a case-by-case basis by the Memorial Sloan Kettering Cancer Center and the Medical University of Vienna.

## Research in context

### Evidence before this study

We searched PubMed for deep learning studies that focused on PET in cancer, including articles published before Oct 30, 2022. Search terms were (“deep learning” OR “machine learning” OR “artificial intelligence”) AND “PET” AND (“cancer” OR “tumor” OR “carcinoma” OR “lymphoma”). No language restrictions were applied to the search. We identified several studies that focused on the development of algorithms for tumour segmentation on [^18^F]fluorodeoxyglucose-PET, and several that focused on PET-based outcome prediction or prognostication, most of them using small-to-moderate numbers of pre-treatment scans. Few smaller-sized studies (fewer than 750 [^18^F]FDG-PET scans) reported on the performance of algorithms for the detection of hypermetabolic tumour sites on a per-lesion level. A single study in different cancers trained a deep-learning algorithm to classify [^18^F]FDG-PET scans, that labelled the scans as positive, negative, or unclear for the presence of hypermetabolic tumour sites, without standardised PET criteria for curation, and without external validation.

### Added value of this study

Our deep learning algorithm, Lymphoma Artificial Reader System, which was trained on four to five times more PET-CT scans as previous studies, could distinguish between [^18^F]FDG-PET-CT scans with and without hypermetabolic tumour sites in patients with lymphoma. Contrary to most previous studies, we included both pre-treatment and post-treatment scans to reflect clinical routine, and we used the well established Lugano PET criteria in combination with previous and follow-up scans and biopsy results to provide a reliable reference standard. Our algorithm demonstrated high levels of accuracy, sensitivity, and specificity in both internal and external test cohorts, despite technical and clinical differences between centres and patient cohorts.

### Implications of all the available evidence

The strong performance of our algorithm suggests that it could potentially be used to rule out hypermetabolic tumour sites post treatment, or as a decision support or second reader tool, especially when [^18^F]FDG-PET-CT scan volumes are high.
