# Supplementary material for: Deep learning for [18F]fluorodeoxyglucose-PET-CT classification in patients with lymphoma: a dual-centre retrospective analysis
Source: Lancet Digit Health. Author manuscript; Available in PMC 2024 Mar 27. (PMC10972536; doi:10.1016/S2589-7500(23)00203-0)
Supplement: 1 [file NIHMS1961979-supplement-1.pdf]

THE LANCET
Digital Health

Supplementary appendix

This appendix formed part of the original submission and has been peer reviewed. We post it as supplied by the authors.

Supplement to: Häggström I, Leithner D, Alvén J, et al. Deep learning for [^{18}F]fluorodeoxyglucose-PET-CT classification in patients with lymphoma: a dual-centre retrospective analysis. *Lancet Digit Health* 2023; published online Dec 21. https://doi.org/10.1016/S2589-7500(23)00203-0.

APPENDIX

Deep learning for [18F]fluorodeoxyglucose-PET-CT classification in patients with lymphoma: a dual-centre retrospective analysis

Ida Häggström[1,2], Doris Leithner[2,3], Jennifer Alvén[1], Gabriele Campanella[4,5], Murad Abusamra[2], Honglei Zhang[2], Shalini Chhabra[2], Lucian Beer[6], Alexander Haug[6], Gilles Salles[7,8], Markus Raderer[9], Philipp B. Staber[7], Anton Becker[2,3,8], Hedvig Hricak[2,8], Thomas J. Fuchs[4,5], Heiko Schöder[2,8], Marius E. Mayerhoefer[2,3,6,8]

[1]Department of Electrical Engineering, Chalmers University of Technology, Gothenburg, Sweden
[2]Department of Radiology, Memorial Sloan Kettering Cancer Center, New York, NY, USA
[3]Department of Radiology, NYU Langone Health, Grossman School of Medicine, New York, NY, USA
[4]Hasso Plattner Institute for Digital Health, Mount Sinai Medical School, New York, NY, USA
[5]Department of AI and Human Health, Icahn School of Medicine at Mount Sinai, New York, NY, USA
[6]Department of Biomedical Imaging and Image-guided Therapy, Medical University of Vienna, Vienna, Austria
[7]Department of Medicine, Memorial Sloan Kettering Cancer Center, New York, NY, USA
[8]Weill Cornell Medical College, Cornell University, New York, NY, USA
[9]Department of Medicine I, Medical University of Vienna, Vienna, Austria

---

Contents

1 Data Preprocessing . . . **2**
Table 1: PET scan attributes of the cohorts . . . 3

2 DNN Model Training . . . **4**
Figure 1: Different LARS variants using PET, CT or PET+CT . . . 6

3 Extended results . . . **7**
Table 2: Classification accuracy by Deauville category . . . 7
Table 3: Missclassification categories . . . 7
Figure 2: Examples of false-positive PET images . . . 8
Figure 3: UMAP visualisation of image features . . . 9
Figure 4: Grad-CAM visualizations . . . 10
Figure 5: Example PET images with large prediction differences between MIP views . . . 11

4 Dataset Size Experiment . . . **12**
Figure 6: AUCs for different amounts of training data . . . 12

5 Integration of Clinical Information into DNN . . . **13**
Figure 7: Performance for combination models using PET + other data . . . 13

References . . . **14**

1. Data Preprocessing

PET-CTs were retrieved from the Picture Archiving and Communication Systems (PACS) at MSK and MUV. All imaging attributes are found in Table 1. Standardized Uptake Values (SUV) for PET were calculated using the equation

$$\mathrm{SUV}\,[\mathrm{g/ml}] = \frac{C\,[\mathrm{Bq/ml}] \cdot D\,[\mathrm{Bq}]}{W\,[\mathrm{g}]} \tag{1}$$

where C is the radioactivity concentration at a given time point, D is the dose at the start of image acquisition (corrected for decay since injection), and W is the patient's body mass.

The majority of PET/CTs were scans from the skull base to mid-thigh, and a small number were head-to-toe scans. All PET images were resampled to an isotropic voxel size of $3.27 \times 3.27 \times 3.27\,\mathrm{mm}^3$. Head-to-toe scans were automatically cropped to approximately the same view as the standard whole-body scans, by identifying the top of the head and including 300 slices down. Based on 3D PET volumes, maximum intensity projections (MIPs), and one coronal and one sagittal 2D MIP image were created, cropped to their respective non-zero bounding box, and clipped to a maximum SUV of 30. The full isotropic 3D PET volumes were also saved using the same procedure. Similarly, the CT images were also resampled to an isotropic voxel size of $3.27 \times 3.27 \times 3.27\,\mathrm{mm}^3$ to match the PET images and cropped using the bounding box obtained from the PET preprocessing.

The MUV cohort was preprocessed in the same way as the MSK cohort. The images from MUV were not post-filtered in any way, and as this is standard at MSK, we post-filtered the MUV images by a Gaussian filter of 6.4 mm according to the MSK clinical standard. The MUV CT images were also preprocessed in the same manner as those from MSK.

There were $n = 85$ (out of total $n = 16583$) CT scans that could not be retrieved in the MSK dataset (broken or missing CT DICOM corresponding to the whole-body PET), of which 73 were training + tuning, and 12 were test dataset scans. Thus, the models using CT alone or the 2-channel PET-CT images (LARS-ct, LARS-ptct) were trained and tested without these 85 samples.

Table 1: PET scan attributes of the 16583 scans in the MSK cohort and 1000 scans in the external MUV cohort.

Attribute	MSK Cohort<br>Training+tuning dataset<br>(n=13258)	Test dataset<br>(n=3325)
PET scanner		
GE Discovery 690	4406	1060
GE Discovery 710	3158	836
GE Discovery STE	3151	783
GE Discovery 600	905	214
GE Discovery MI	816	222
GE Discovery ST	634	155
Siemens Biograph 1080	124	39
GE Discovery LS	48	14
GE SIGNA PET/MR	3	0
NA	13	2
Injected dose (MBq), median (IQR)	282.8 (261.7–299.7)	284.1 (262.0-300.2))
Patient weight (kg), median (IQR)	76 (63–89)	76 (65, 91)
Voxel size (mm x mm x mm), mean±SD	5.5±0.1 x 5.5±0.1 x 3.3±0.2	5.5±0.1 x 5.5±0.1 x 3.3±0.2
**Attribute**	**MUV Cohort**	**Test dataset<br>(n=1000)**
PET scanner		
Siemens Biograph64 Truepoint		992
Siemens Biograph128 Vision 600 Edge		5
Siemens Biograph 1080		2
Siemens Biograph40 mCT		1
Injected dose (MBq), median (IQR)		198.5 (181.3–214.2)
Patient weight (kg), median (IQR)		74 (62–85)
Voxel size (mm x mm x mm), mean±SD		4.1±0.1 x 4.1±0.1 x 3.0±0.1

2. DNN Model Training

We trained our DNN model LARS on the internal training dataset (80% of MSK scans) to classify scans as positive or negative for FDG-avid tumor sites. We used a ResNet34 convolutional neural network (CNN) for feature extraction[1] with an input image size of $1 \times 310 \times 310$ for 2D MIPs, and $c \times 250 \times 250 \times 300$ for 3D models, where the number of channels c was equal to 1 for individual PET and CT models and equal to 2 for the joint PET/CT model. The output was always 512 features (ResNet34). The images were weakly labeled (negative=0, or positive=1 for hypermetabolic tumor sites), without further annotations. Additional feature extractor networks were also explored, including the convolutional networks ResNet501, ShuffleNet2[2], DenseNet121[3], and 169[3], and a vision transformer network ViT[4]. Experimentally, the best performance was obtained using ResNet34, and therefore, this architecture was selected for further experiments.

During training and testing, all input images were first normalized. 2D and 3D PET datasets were clipped at SUV 30 and z-normalized. The CT dataset was clipped at -125 HU and +225 HU, followed by [0,1] normalization.

For augmentation during training, each PET MIP was randomly positioned within an empty 310×310 canvas and used as a 1-channel input to the DNN. Similarly, the 3D input PET and/or CT volume was randomly positioned on a $250 \times 250 \times 300$ canvas. Five additional augmentation techniques were investigated at training time: 1) random left-right-up-down flips, 2) random 90° rotations, 3) random left-right flips, 4) random ±15% pixel value (SUV) scaling, and 5) random Gaussian noising with σ between 0.01 and 0.1, and different combinations of the aforementioned augmentations. The only augmentation technique that improved the validation results on any LARS variant was the random SUV scaling for PET MIPs (LARS-avg and LARS-max), which is why this augmentation was applied during training for those models.

We constructed seven LARS variants:

- LARS-avg: classification based on the average (mean) probability of coronal and sagittal 2D PET MIP images, aiming for high accuracy.
- LARS-max: classification based on the maximum probability of coronal and sagittal 2D PET MIP images, aiming for high sensitivity.
- LARS-pt3d: classification based on the probability of the full 3D PET volume.
- LARS-ct: classification based on the probability of the full 3D CT volume.
- LARS-ptct: classification based on the probability of the full 3D PET/CT volume.
- LARS-avg-ct: classification based on the probability of the separately trained LARS-avg aggregated with the probabilities of LARS-ct.
- LARS-max-ct: classification based on the probability of the separately trained LARS-max aggregated with the probabilities of LARS-ct. Note that this uses no new training.

Note that LARS-avg and LARS-max stemmed from the same trained model but with different prediction aggregation. For a schematic overview of trained models, see Figure 1.

The training dataset was bootstrapped (randomly split with replacement) 20 times at the patient level into 80% and 20% training and tuning subsets, respectively, and a separate model was trained on each split. The final ensemble model consisted of the 10 best-performing models (top-10), where all predictions were averaged into a single prediction per scan.

The DNN was trained using a binary cross-entropy (BCE) loss function, and both stochastic gradient descent (SGD) with mini-batches and the Adam optimizer[5] were tested, and the mini-batch size was set to 100 for 2D models and four for 3D models. Learning rates ranged in powers of 10 from 0.00001 to 0.1. The settings that yielded the best average tuning set performance (i.e., the highest AUC) were chosen, in this case, 0.01 using SGD. We trained the DNN models until convergence using an early stopping scheme. The model was implemented and trained using PyTorch (v1.9)[6] on a high-performance computer cluster of Nvidia DGX-1, GTX 1080, and GTX 1080Ti graphics processing units (GPUs). Each training run took approximately ten hours for an average of nine epochs.

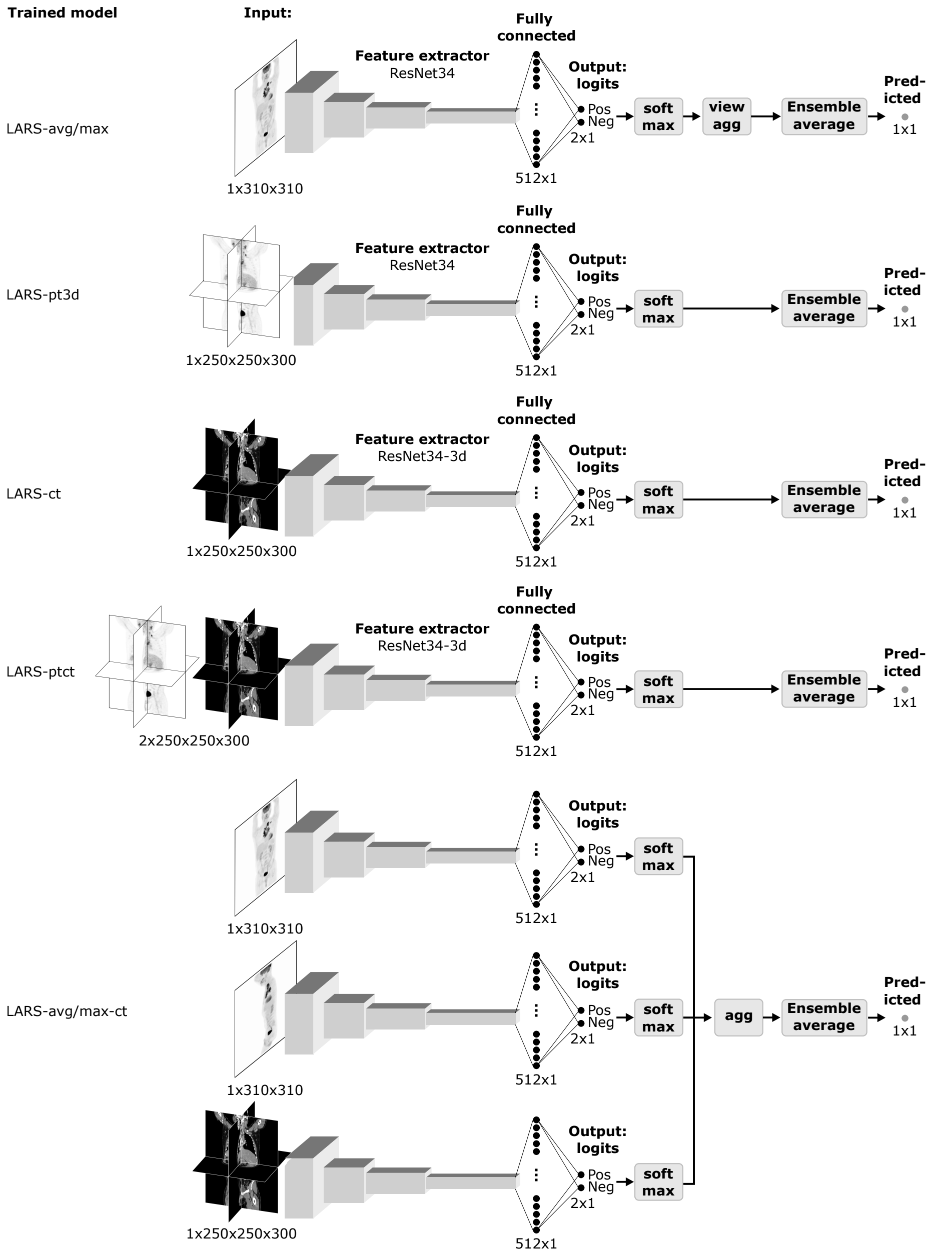

Figure 1: Overview of different LARS variants. LARS-avg and LARS-max stem from the same trained model, but differ by aggregation of prediction values for the two MIP views. Similarly, LARS-avg-ct and LARS-max-ct are based on the trained LARS-avg/max models and the trained LARS-ct model, where the mean or max MIP view aggregated prediction from LARS-avg/max is aggregated with the single prediction from LARS-ct. LARS-pt3d, LARS-ct and LATS-ptct are all 3D models based on a 3D ResNet34. LARS-ptct is a 2-channel model utilizing the full 3D PET and 3D CT image data.

3. Extended results

Tables 2 and 3 show the classification accuracy per Deauville category and the misclassification categories labeled by a senior rater, respectively.

Figure 2 show eight examples of false-positive cases for LARS-avg with uptake not related to lymphoma. Figure 3 visualizes extracted image features using UMAP, and Figure 4 shows Grad-CAM heatmaps for three examples of true-positive cases. Finally, Figure 5 shows examples of cases with large prediction differences between the two MIP-views for LARS-avg.

Table 2: Test dataset (n=3316 for MSK, n=993 for MUV) classification accuracy of LARS variants by Deauville category. Results are shown after the removal of incorrectly labeled cases.

		LARS Variants						
	% of cases	LARS-avg	LARS-max	LARS-pt3d	LARS-ct	LARS-ptct	LARS-avg-ct	LARS-max-ct
**MSK:**								
Deauville 1	31.1	92.4	84.0	92.9	58.9	93.5	91.8	82.9
Deauville 2	12.4	89.5	81.2	92.0	59.7	91.2	88.5	83.9
Deauville 3	11.2	90.5	80.4	90.5	56.5	90.5	88.6	87.0
Deauville 4	11.2	59.4	69.8	50.3	66.0	55.0	61.2	97.6
Deauville 5	34.0	96.5	98.4	91.7	67.8	93.3	96.4	99.9
**MUV:**								
Deauville 1	25.1	94.5	92.8	93.2	79.2	95.2	92.8	98.0
Deauville 2	15.6	92.9	86.5	87.7	87.1	91.0	89.7	96.8
Deauville 3	15.0	95.2	89.1	91.2	85.0	88.4	91.8	98.0
Deauville 4	14.8	67.3	73.5	58.5	91.8	61.2	70.1	99.3
Deauville 5	29.5	99.0	99.3	94.2	92.2	94.2	98.6	100.0

Table 3: Categories of misclassification for incorrectly predicted scans of the MSK test dataset (n=358) and the external MUV test dataset (n=89) for LARS-avg.

	Prediction group			
	False positive		False negative	
Error class	MSK	MUV	MSK	MUV
Incorrect labeling	1	2	8	5
Infection / inflammation	70	12	7	0
Iatrogenic	30	6	4	1
Brown fat	7	0	3	1
Other	28	6	1	0
Unknown	22	7	177	49

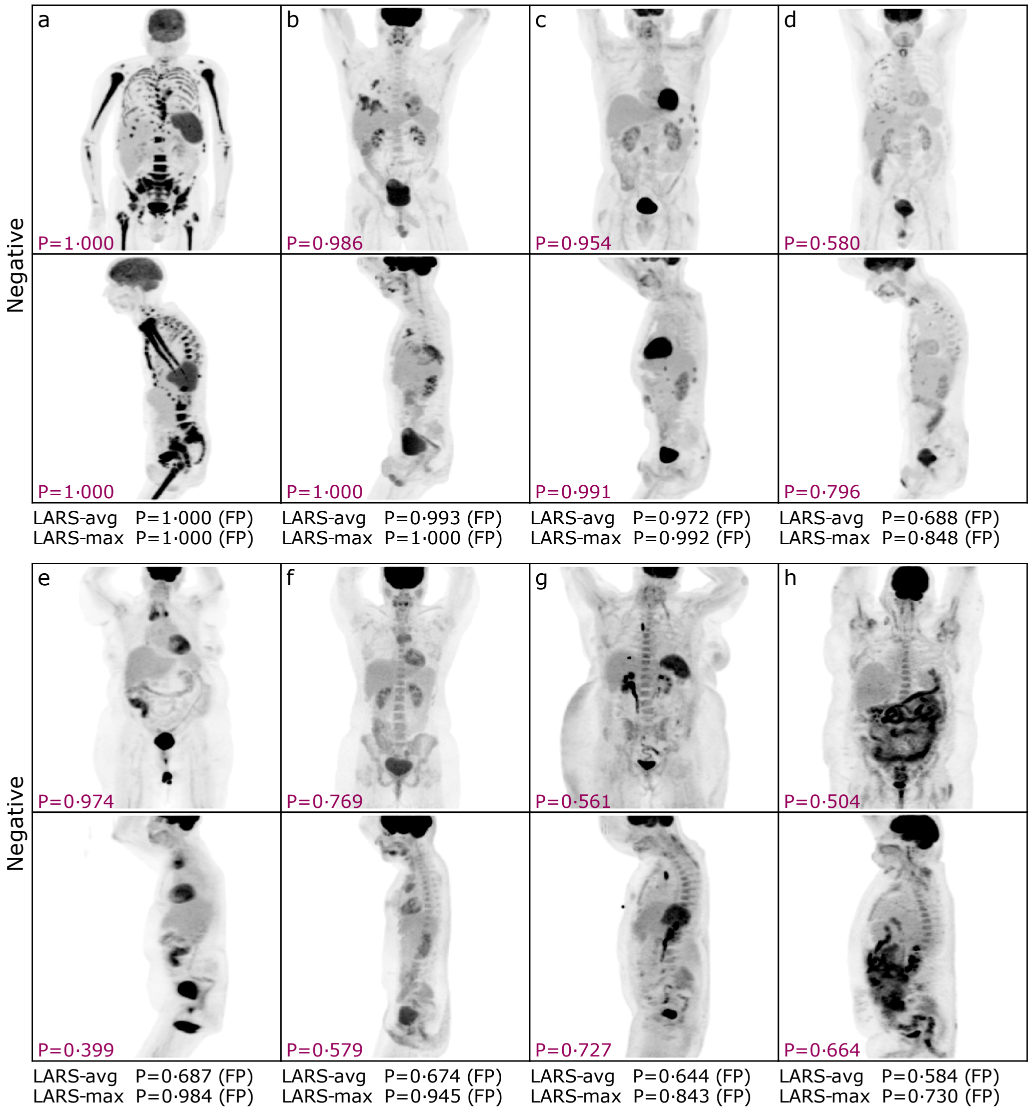

Figure 2: Examples of false-positive (FP) cases with areas of increased [18F]FDG uptake on PET not related to lymphoma: G-CSF treatment-related marked bone marrow and spleen repopulation/activation (a) (category: iatrogenic); right lung pneumonia (b) (category: infection/inflammation); left lower rib fractures (s) (category: other); right lung pleurodesis-related uptake (d) (category: iatrogenic); thyroiditis (e) (category: infection/inflammation); post-treatment thymic rebound (f) (category: iatrogenic); port-a-cath / catheter thrombus-related uptake (g) (category: iatrogenic); marked bowel uptake due to metformin intake (h) (category: iatrogenic); The SUV range of all panels is 0–10. p: predicted probability of hypermetabolic tumor sites.

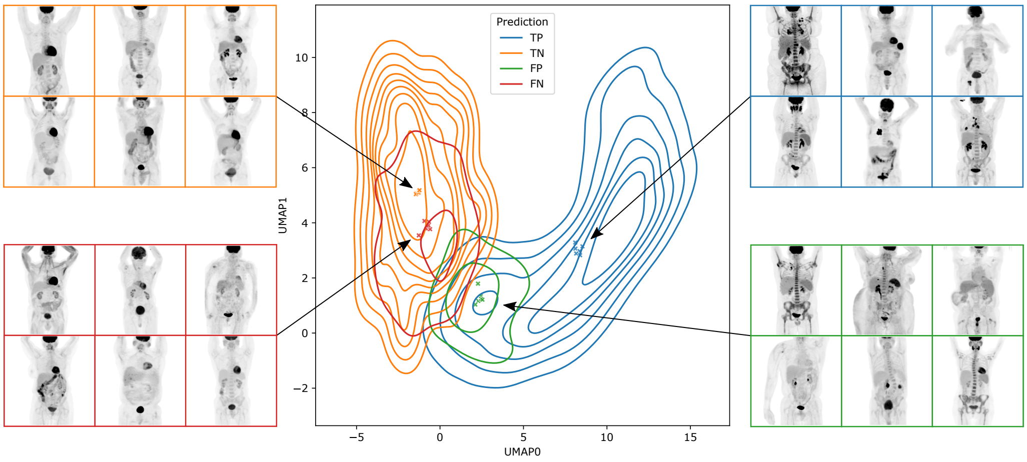

Figure 3: Visualization of the extracted image features of the internal MSK test dataset (n=3325). The 512 image features were reduced to two using UMAP. Each image constitutes one data point, and isolines of the distribution are shown rather than individual markers to improve visibility. For each prediction class (TP: true-positive; TN: true-negative; FP: false-positive; FN: false-negative), the six images closest to their respective class average feature are shown as thumbnails.

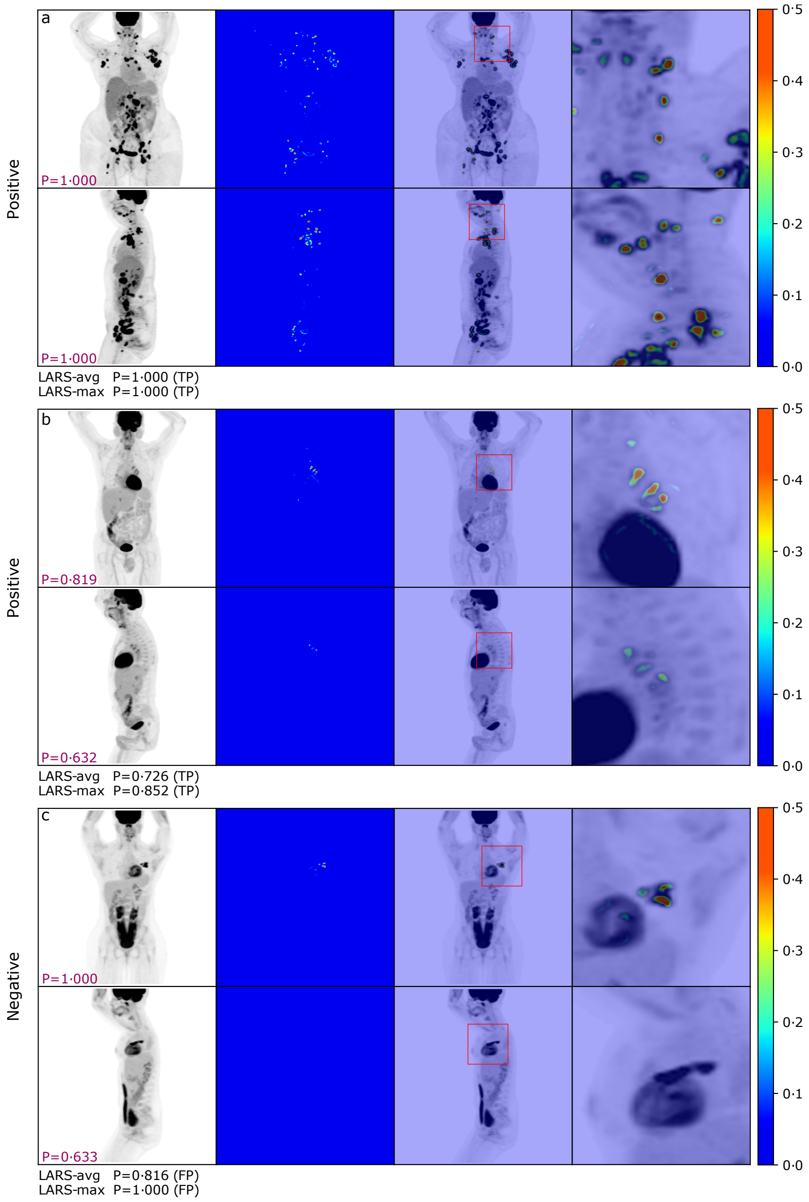

Figure 4: Model performance visualization using Grad-CAM. Each panel shows the original image (left; SUV range, 1–10), Grad-CAM heatmap (2nd left), overlay image (2nd right), and magnified region of high importance (right) on coronal and sagittal MIP views. Higher heatmap values (red) indicate greater importance for positive prediction. Hypermetabolic tumor sites in true-positive (TP) cases are well visualized by Grad-CAM (a, b). While the physiologic uptake in the abdominal muscles is correctly ignored by Grad-CAM (c), the left lung biopsy-proven necrotizing pneumonia was regarded as probable tumor by Grad-CAM, consistent with the false-positive (FP) prediction by LARS. p: predicted probability of hypermetabolic tumor sites.

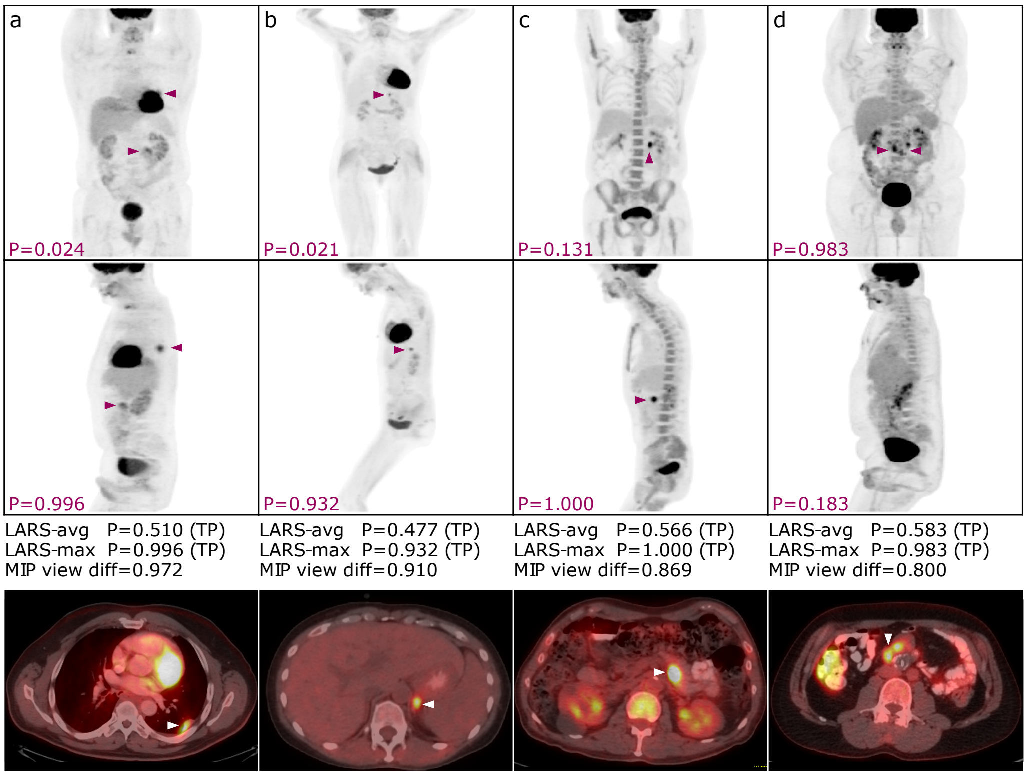

Figure 5: Four true- positive (TP) cases from the MSK test set (n=3325) with a large absolute difference between the predicted probabilities (p) on the sagittal and coronal MIP views. Panels are labeled with ensemble prediction class as well as the respective view probability (averaged over the top-10 models), and the absolute difference between the two views. The SUV range of all panels is 0–10. Probability differences are due to physiological [18F]FDG uptake that obscured lesion [18F]FDG uptake (arrowheads), or lesion uptake that mimicked physiologic uptake: in (a), left pleural lesion uptake obscured by cardiac uptake, and left paraaortic lymph node uptake obscured by renal pelvis tracer accumulation on the coronal view; in (b) left adrenal gland lymphoma manifestation mimics left upper pole renal tracer excretion on the coronal view, but is shown to be located more anteriorly on the sagittal view; in (c), duodenojejunal flexure lymphoma manifestation uptake mimics left renal pelvis tracer excretion on the coronal view, but is distinct on the sagittal view; in (d), paraaortic nodal lymphoma uptake is partly obscured by, and partly mimics left renal tracer excretion. Color-coded fused PET-CT images (lower row) show sites of the actual lesions.

4. Dataset Size Experiment

To evaluate the impact of the size of the training dataset on classification, we performed six additional experiments with the training dataset subsampled from 100% of the cohort size (13258 PET scans) to 50% (6714 PET scans), 20% (2627 scans), 10% (1348 scans), 5% (680 scans), 2% (268 scans), and 1% (125 scans), respectively, for each of the 20 data splits using LARS-avg. The allocation of each patient to the training or tuning dataset was predetermined for all of the 20 data splits and remained fixed. After training the 20 models for each of the six subsampled datasets, we also calculated the top-10 ensemble prediction. Results show that model performance as measured by AUC increased with increasing training dataset size, from 0.74 at 1% to 0.95 at 100% (see appendix Figure 6). The model performance appeared to start leveling off at the full dataset size (n=13258), although it did not completely plateau, and additional training data would likely further improve results. The model performance at 2–5% of the training dataset size (n=268 to 680 scans) represents the typical size seen in the literature[7,8,9]. Based on our experiments, one can assume that these models may not have reached their full potential. Clearly, large amounts of diverse training data are necessary to develop generalizable and reliable DNN models. Newer methods utilizing the concept of self-supervision may ameliorate the limited performance when little training data is available.

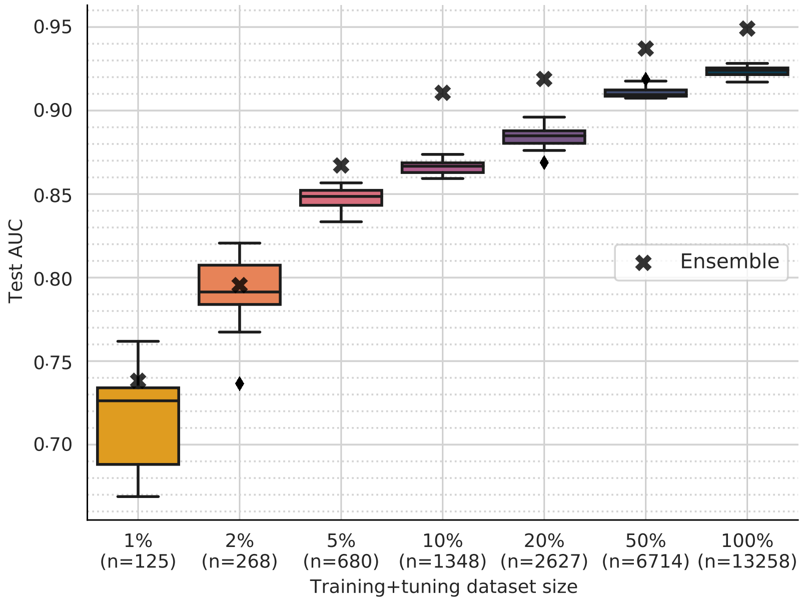

Figure 6: Areas under the curve (AUC) of the MSK test dataset (n=3325) for different sizes of the training+tuning data, using LARS-avg. Ensemble results are shown as crosses. The boxes represent the 20 trained models. Bonferroni corrected pairwise t-tests confirmed significant AUC differences between data set sizes (p<0.001).

5. Integration of Clinical Information into DNN

To determine whether the classification performance of LARS could be improved by other data that potentially facilitate the differentiation between lymphoma-associated and non-neoplastic [18F]FDG uptake, we incorporated the following parameters into the model: age as a continuous variable; chemo(immuno)therapy within 30 days prior to the PET scan as a binary variable; and G-CSF treatment within 30 days prior to the PET scan as a binary variable. The rationale for including age was the age-dependent prevalence of degenerative joint disease, which frequently shows increased uptake on PET [10]. Chemo(immuno)therapy may lead to treatment-related inflammatory [18F]FDG uptake, whereas G-CSF treatment frequently leads to increased bone marrow [18F]FDG uptake due to marrow activation/repopulation, and also to increased splenic uptake [11]. The 1D array of variables were fully connected to an output of the same size as the feature vector of the image CNN (512) and added to the image features before the final fully connected layer with binary output. The AUCs of the tuning sets for the DNN models combining PET images with clinical information are shown in appendix Figure 7; the additional information did not improve the performance over the model using only PET images, and therefore, this approach was not investigated further.

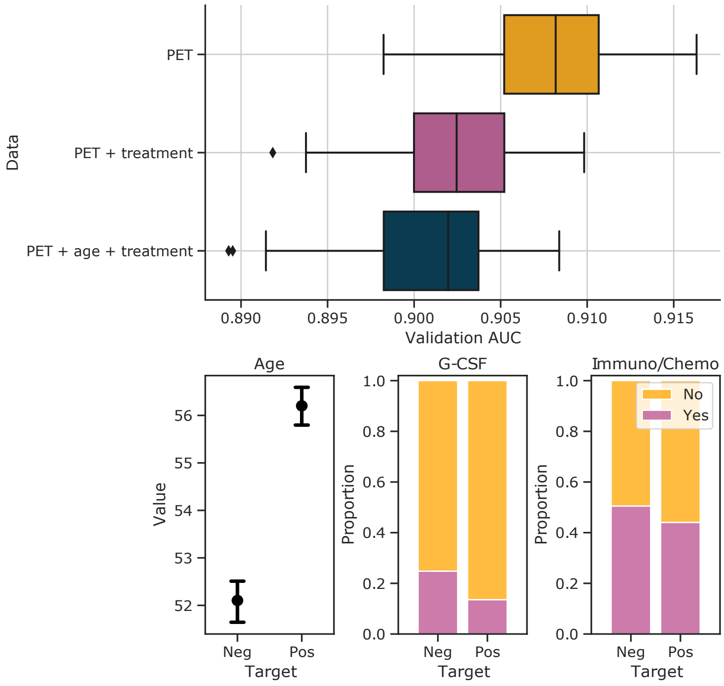

Figure 7: Comparison of deep neural network models using only PET images, PET images plus treatment information, or PET images plus treatment and age information. Box plots in the upper row show areas under the curve (AUC) of the respective validation sets for the 20 models (for the 20 data splits). Lower row box plot and bar charts show average values of age, G-CSF, and immuno-/chemotherapy for negative and positive cases of the full MSK cohort.

References

1 He, K., Zhang, X., Ren, S., & Sun, J. (2016). Deep residual learning for image recognition. Proceedings of the IEEE Computer Society Conference on Computer Vision and Pattern Recognition, 770–78.

2 Ma, N., Zhang, X., Zheng, H. T., & Sun, J. (2018). Shufflenet V2: Practical guidelines for efficient CNN architecture design. Lecture Notes in Computer Science, 122–38.

3 Huang, G., Liu, Z., Van Der Maaten, L., & Weinberger, K. Q. (2017). Densely connected convolutional networks. Proceedings of the IEEE Conference on Computer Vision and Pattern Recognition, 2261–9.

4 Dosovitskiy, A., Beyer, L., Kolesnikov, A., et al. (2021). An Image is Worth 16x16 Words: Transformers for Image Recognition at Scale. International Conference on Learning Representations (ICLR) 2021. Available at: http://arxiv.org/abs/2010.11929.

5 Kingma, D. P., & Ba, J. (2014). Adam: A Method for Stochastic Optimization. arXiv. 2014; 1412.6980. Available from https://arxiv.org/abs/1412.6980

6 Paszke, A., Gross, S., Massa, F., et al. (2019). PyTorch: An Imperative Style, High-Performance Deep Learning Library. In: Wallach H., Larochelle H., Beygelzimer A., d'Alché-Buc F., Fox E., Garnett R., editors. Advances in Neural Information Processing Systems 2019; 32: 8024–35.

7 Sibille, L., Seifert, R., Avramovic, N., et al. (2020). ^{18}F-FDG PET/CT Uptake Classification in Lymphoma and Lung Cancer by Using Deep Convolutional Neural Networks. Radiology, 294, 445–52.

8 Blanc-Durand, P., Jégou, S., Kanoun, S., et al. (2021). Fully automatic segmentation of diffuse large B cell lymphoma lesions on 3D FDG-PET/CT for total metabolic tumour volume prediction using a convolutional neural network. Eur J Nucl Med Mol Imaging, 48, 1362–70.

9 Jiang, C., Chen, K., Teng, Y., et al. (2022). Deep learning–based tumour segmentation and total metabolic tumour volume prediction in the prognosis of diffuse large B-cell lymphoma patients in 3D FDG-PET images. Eur Radiol, 32, 4801–12.

10 Nguyen, B. J., Burt, A., Baldassarre, R. L., et al. (2018). The prognostic and diagnostic value of $^{18}F\text{-}FDG$ PET/CT for assessment of symptomatic osteoarthritis. Nucl Med Commun, 39, 699–706.

11 Minamimoto, R., Baratto, L., Iagaru, A. (2022). Association Between Time Since Administration of Pegylated G-CSF (Pegfilgrastim) and Bone Marrow Uptake on FDG PET-CT: Determination of a Minimum Interval. AJR Am J Roentgenol, 218, 351–8.
